# Finding and identifying the viral needle in the metagenomic haystack: trends and challenges

**DOI:** 10.3389/fmicb.2014.00739

**Published:** 2015-01-07

**Authors:** Hayssam Soueidan, Louise-Amélie Schmitt, Thierry Candresse, Macha Nikolski

**Affiliations:** ^1^Bordeaux Bioinformatics Center, Université de BordeauxBordeaux, France; ^2^INSERM U1035, Université de BordeauxBordeaux, France; ^3^Centre National de la Recherche Scientifique/Laboratoire Bordelais de Recherche en Informatique, Université de BordeauxTalence, France; ^4^Institut National de la Recherche Agronomique, UMR 1332 Biologie du Fruit et PathologieVillenave d'Ornon, France; ^5^UMR 1332 Biologie du Fruit et Pathologie, Université de BordeauxVillenave d'Ornon, France

**Keywords:** microbial metagenomics, NGS, virome, host—pathogen interactions, taxonomic assignment

## Abstract

Collectively, viruses have the greatest genetic diversity on Earth, occupy extremely varied niches and are likely able to infect all living organisms. Viral infections are an important issue for human health and cause considerable economic losses when agriculturally important crops or husbandry animals are infected. The advent of metagenomics has provided a precious tool to study viruses by sampling them in natural environments and identifying the genomic composition of a sample. However, reaching a clear recognition and taxonomic assignment of the identified viruses has been hampered by the computational difficulty of these problems. In this perspective paper we examine the trends in current research for the identification of viral sequences in a metagenomic sample, pinpoint the intrinsic computational difficulties for the identification of novel viral sequences within metagenomic samples, and suggest possible avenues to overcome them.

## Introduction

While genomics is the research field relative to the study of the genome of any organism, metagenomics is the term coined for the research that focuses on many genomes at the same time, as typical in some sections of environmental studies. The analysis of microbial communities has been until recently a complicated if not untractable task due to their high diversity and to the fact that many of these organisms cannot be cultured. Harnessing the major advances achieved in sequencing technologies, metagenomics has emerged as the only currently available approach to extensively characterize these largely unculturable communities. Besides vastly enriching our knowledge of microbial diversity in a varied range of environments, and providing information on the dynamics and on the overall functioning of microbial communities, metagenomics is also shedding light on many important biological processes and, in particular, on the role of the microbiome in biological functions essential for the development of higher order organisms harboring it (Blottière et al., 2013; Manor et al., 2014), or in the development of pathological problems (Cénit et al., 2014; Vayssier-Taussat et al., 2014). In addition, metagenomic efforts also vastly enrich the repertoire of genes available for biotechnological applications (Ni and Tokuda, 2013).

At the same time metagenomics extensively relies on bioinformatics to tackle the huge amounts of sequence data involved, and recognizes the need to develop computational methods that maximize our understanding of the genetic composition and the biological activities expressed in communities so complex that they can only be sampled, never completely characterized. Computational analysis has become a genuine bottleneck for metagenomics due not only to the large amount of sequence data, but also to the new questions such as, for example, the need for simultaneous assembly of multiple genomes or transcriptomes and the analysis of complex networks of host-microbe interactions (Wooley and Yuzhen, 2009).

In this context the analysis of viral communities presents particular interest but also computational challenges. The ability to thoroughly analyze the viral composition of an environmental sample is of paramount importance, in particular because viruses have turned out to play a major role in the functioning of microbial communities by processes such as viral infection and selective killing of certain taxa or as vectors for horizontal gene transfer (Suttle, 2007). Consequently, the viral part of the microbiome has been shown in a number of situations to have a major impact on the dynamics and on the evolutionary processes of their host populations. The discovery and classification of novel viral species, but also of higher order taxa, is therefore of particular interest in this context (Rosario and Breitbart, 2011).

One of the main goals of metagenomic projects is to characterize the microbial communities in terms of the identity and diversity of species present (species richness) in a given environment. When it comes to species identification, the task is called taxonomic assignment. Current NGS technologies have provided an opportunity for doing this analysis routinely (Petrosino et al., 2009). Software tools for automated taxonomic assignment for organisms such as bacteria and fungi have since become a mature technology and are now routinely used in many studies.

If bacterial or fungal applications have recently seen major advances, the problem of taxonomic assignment for viruses—such as it arises in environmental studies—remains largely unsolved from the computational point of view, as exemplified by the difficulty of distinguishing viral genomes from eukaryotes and bacteria observed in some studies (Bazinet and Cummings, 2012). Indeed, ab-initio identification of a sequence as belonging to a cellular organism or to a virus remains a complicated task outside of the popular sequence-homology based approaches that rely on direct comparisons with already known viral sequences present in international databases.

We distinguish the task of deciding to which first-level domain (eukaryotes, bacteria, archaea, virus) a given sequence belongs—that we call first-level assignment—from a more fine-grained taxonomic assignment at, e.g., family, genus or species level. In virome studies the latter task is greatly facilitated when targeted sequencing of purified viral particles is performed (Hall et al., 2014), but the former is particularly difficult for complex samples containing both eukaryotic and viral sequences and when, as is very frequently the case, unknown viral species are present. In this paper we examine reasons behind this difficulty and suggest possible avenues to overcome them.

## First-level classification of complex environmental samples

The first-level assignment of sequence data coming from a non-targeted sequencing of a metagenomic sample is a particularly challenging computational problem. The most blatant difficulty is in the recognition of novel viral sequences, for which no close homologs have been previously characterized. This question is however of paramount importance for the biologists. From the biodiversity point of view, the identification of unknown viruses representing novel higher order taxa (genera, families…) is of clear interest as evidenced, for example, by the discovery of the Mimiviruses with genomes exceeding in size those of many bacterial genomes (Claverie et al., 2009). But this question can also have important practical implications as when trying to identify novel viruses responsible for particular syndromes or diseases in humans, plants or husbandry animals (Roossinck, 2012; Lecuit and Eloit, 2014).

A number of bioinformatics methods efficiently perform the first-level assignment of sequences from a sample mainly containing known species. Computational solutions can be broadly organized in two main categories: (1) sequence similarity methods and (2) sequence composition methods.

Methods that rely on sequence similarity can be themselves subdivided in alignment-based techniques (mostly attempting to improve BLAST accuracy) and index-based. Alignment-based methods suffer from two limitations: speed and lack of sensitivity (e.g., Bazinet and Cummings, 2012; Wood and Salzberg, 2014). Recently, novel solutions have been suggested to overcome these limitations. These methods are based on long *k*-mers (words of size *k*) and conceptually rely on the fact that when *k* is sufficiently large, *k*-mers become very specific. Consequently, the idea is to index the databases by long *k*-mers. This is indeed the foundation of MegaBlast (a general-purpose sequence aligner using long seeds), but also of a number of methods specific for taxonomic assignment such as LMAT (Ames et al., 2013) and Kraken (Wood and Salzberg, 2014). The downside of these approaches is over-specificity, which makes classification of unknown sequences problematic. This limitation can be particularly acute given the known very high intraspecific variability existing in some viral species or higher order taxa. For example, current criteria of the International Committee for the taxonomy of viruses tolerate up to 28% of nucleotide sequence divergence for the polymerase or capsid protein genes for isolates of a same species in the *Betaflexiviridae* family and a similar level of divergence at the whole genome level in the *Potyviridae* family (King et al., 2012).

A complementary approach is based on sequence composition analysis. Such methods rely on the decomposition of sequences into frequencies of short *k*-mers and make use of machine learning techniques (e.g., SVM, kNN, Naive Bayes, etc.) to train a classifier on a reference database. The taxonomic assignment of novel sequences is then predicted by applying the pre-trained model. These methods theoretically are better suited to the task of novel species classification as short *k*-mers distributions are less prone to over-fitting. However, even these techniques fail to classify about 50% of species absent from the training set (Nalbantoglu et al., 2011). This is especially salient for viral sequences, as the vast majority of them fail to be uniquely assigned to any domain of life (Rosen et al., 2010).

Recent results show that *contig*-level assembly improves the strength of the taxonomic signal contained in individual short reads, even in the case of increased chimericity (Mende et al., 2012; Teeling and Glockner, 2012). This is why in our experimental evaluation (see Section Why is the First-level Assignment Problem Hard?) we work exclusively with sequence lengths that are comparable to contigs obtained by a standard metagenomic assembly step when the data originate from complex biological communities.

In summary, even the simple goal to provide a first-level description of a sample composition and be able to reveal if viral sequences are present, has been eluding a satisfactory solution. Indeed, for viral (and also eukaryotic) sequences, none of the existing methods produces a taxonomic distribution that is even remotely close to the expected one (Bazinet and Cummings, 2012).

## Fine-grained classification for bacterial and viral communities

On the other side of the spectrum, the problem of fine-grained characterization of datasets produced by targeted sequencing has seen great progress in recent years. Contrary to the analysis of non-selected and therefore more complex metagenomic samples, efficient methods have been developed for cases where certain components of microbial communities are experimentally targeted (bacterial or viral). This has been an effective way to circumvent the difficulty of the first-level assignment, albeit without solving it.

For bacterial communities the most efficient solution is to perform a tag survey, where only partial genomic information is used and the sequencing is performed for marker genes, such as 16S rRNA for prokaryotes and 18S rRNA for eukaryotes (fungi). This simplifies the analysis for two reasons. First, the amount of data remains reasonable (for a high-throughput analysis) and second, known marker genes' taxonomic classification is available through reference taxonomies such as RDP (Cole et al., 2009) or Greengenes (McDonald et al., 2012). Sequence similarity techniques combined with reference taxonomies recapitulate the known distribution of bacterial phyla extremely well (Bazinet and Cummings, 2012). However, this type of analysis has one major pitfall: it does not provide a reliable method to quantify the identified species (Roux et al., 2011).

While this approach is feasible for bacterial populations, it is not applicable for the analysis of viral communities due to the absence of such marker genes (e.g., Edwards and Rohwer, 2005). Virome studies concentrate on the viral part of the environmental sample and isolate viral genomes encapsidated in viral particles that are purified by a combination of filtration and (utlra)centrifugation. This now popular approach drastically reduces the complexity of the community, which makes it possible to assemble longer contigs routinely (10 kb and more), and even complete genomes from low-complexity samples (Coetzee et al., 2010; Minot et al., 2012). However, it does not really solve the problem of first-level assignment but merely sidesteps it: given the purification step, all generated sequences are generally considered “by definition” as viral, unless proven otherwise by homology-based approaches. In addition, this strategy is not without some caveats (see for more details Fancello et al., 2012). For example, the purified particles may contain cellular genome fragments rather than viral genomes, because of the presence of GTA (Lang and Beatty, 2007) or as a consequence of generalized transduction (for a review see Frost et al., 2005). Also, while 0.22 μ filtering avoids contamination by bacterial, archaeal or eukaryotic cells, other DNA-containing elements, such as bacterial vesicles (Biller et al., 2014) may co-purifiy with virions. Such filtering-based purification also excludes the largest viruses and therefore results in an incomplete picture of viral diversity. Moreover, both LA (see Duhaime et al., 2012) and MDA amplifications have their downfalls. For the former, adapter ligation is only possible for dsDNA viruses and hence ssDNA viral genomes are mostly absent in the sample. For the latter, the amplification is preferentially performed for circular ssDNA viruses rather than dsDNA. The effect of the presence of cellular genes on the bioinformatics analysis of viral metagenomic data has been described and some approaches to detect their presence have been proposed (Roux et al., 2014).

Notwithstanding, virome studies have seen large success. In contrast with bacterial communities, alignment-based methods do not seem to be best suited for viral classification. Indeed, as mentioned by Suttle (2007) even for relatively long viral reads the homolog frequency between these reads and protein sequences within the Genbank database is only about 30%. The idea is to avoid the strong sequential constraint imposed by alignment methods on nucleotides' similarity and to capture a global similarity signal based on sequence composition (*k*-mers). Composition-based techniques seem to provide satisfactory results for fine-grained taxonomic classification of filtered viral samples (e.g., Yang et al., 2005; Trifonov and Rabadan, 2010).

## Why is the first-level assignment problem hard?

As we observed in the previous sections, methods for first-level and fine-grained assignment of metagenomic samples co-exist, but exhibit drastically different performances. This naturally raises the question of reasons underlying this performance gap. Since the characterization of metagenomic samples can be formulated as a supervised machine-learning task, we propose here to employ data complexity and hardness measures to compare the intrinsic difficulty of classifying metagenomic samples at the first-level with that of fine-grained assignment.

We consider here three classification tasks whose goals are to assign a class label to each instance of a set of sequences. The three tasks we describe vary by the composition of the set of sequences and by the scope of the class labels to assign.

1. Given a sample of bacterial sequences, to assign each of them to a phylum (e.g., *Proteobacteria*) or to a class (e.g., *Gammaproteobacteria*);
2. Given a sample of viral sequences, to assign each of them to a group (e.g., dsDNA) or to a family (e.g., *Plasmaviridae*); and
3. Given a sample of sequences, to assign each of them to a first-level domain (e.g., bacteria, archaea, eukaryota, or virus).

The classification tasks (1) and (2) are fine-grained assignment problems and mimic characterization of targeted metagenomic studies; while task (3) represents a first-level assignment and mimics the analysis of complex, untargeted environmental samples. Since we are interested in the identification of novel species in large metagenomic samples, we adopted the representation of sequences as *k*-mer frequency vectors.

We analyzed these three classification tasks using an instance-level analysis of data complexity. In supervised machine learning, the performance of a classifier is dependent both on the learning algorithm (e.g., SVM or Naïve Bayes) and on the training data. While global metrics recapitulate overall performances of a classifier, they fail to indicate whether moderate performances are a consequence of wrong parameter adjustments, biased resampling for training data or of the intrinsic difficulty of the classification task. However, recent literature on instance misclassifications demonstrates that for a given classification task, some instances are intrinsically hard to classify and that their presence is indicative of the global difficulty (see Smith et al. (2014) for a review). Most studies agree on the hardness of outlier instances or on instances belonging to a minority class, but Smith demonstrated that simple metrics can actually quantify the intrinsic hardness of an instance. One of these metrics is the *k*-Disagreeing Neighbors (kDN), which measures for a given instance the number of *k* nearest neighbors that do not share its class label. Smith demonstrated that the kDN measure is strongly positively correlated with the misclassification of an instance over a wide range of learning algorithms and of training data resampling.

To compare the classification hardness of the three tasks, we generated from a representative subset of sequenced organisms from Genbank (September 2014 download, 25,624 bioprojects, 100% of viruses, archaea and bacteria, 24 eukaryotes with 18 plants) 100 sets of 10,000 randomly chosen contiguous genomic fragments of 500 nt average length (corresponding to the average size of metagenomic contigs to simulate an assembly step). For task (1), only bacterial genomes were considered, for task (2) only viral genomes were considered, while for task (3) a balanced composition of viruses, archaea, bacteria and eukaryotes were considered. Each sequence was represented as a 3-mer frequency vector (i.e., the number of time each possible 3-nt sub-sequence appears in the contig) and we defined the distance between two contig as the Euclidean distance between their respective 64 (4^3^) dimensional vectors. For each contig, its kDN value is the number of other contigs that do not share its class label among its closest 73 neighbors. The corresponding class hardness is then measured as the median kDN of all the contigs in a given class. We also determined whether an observed median kDN is significantly extreme (low value indicating easy classification, high value corresponding to difficult classes), by estimating the distribution of the median kDN under the null hypothesis of no relation between class labels by random permutations.

**Figure 1:**
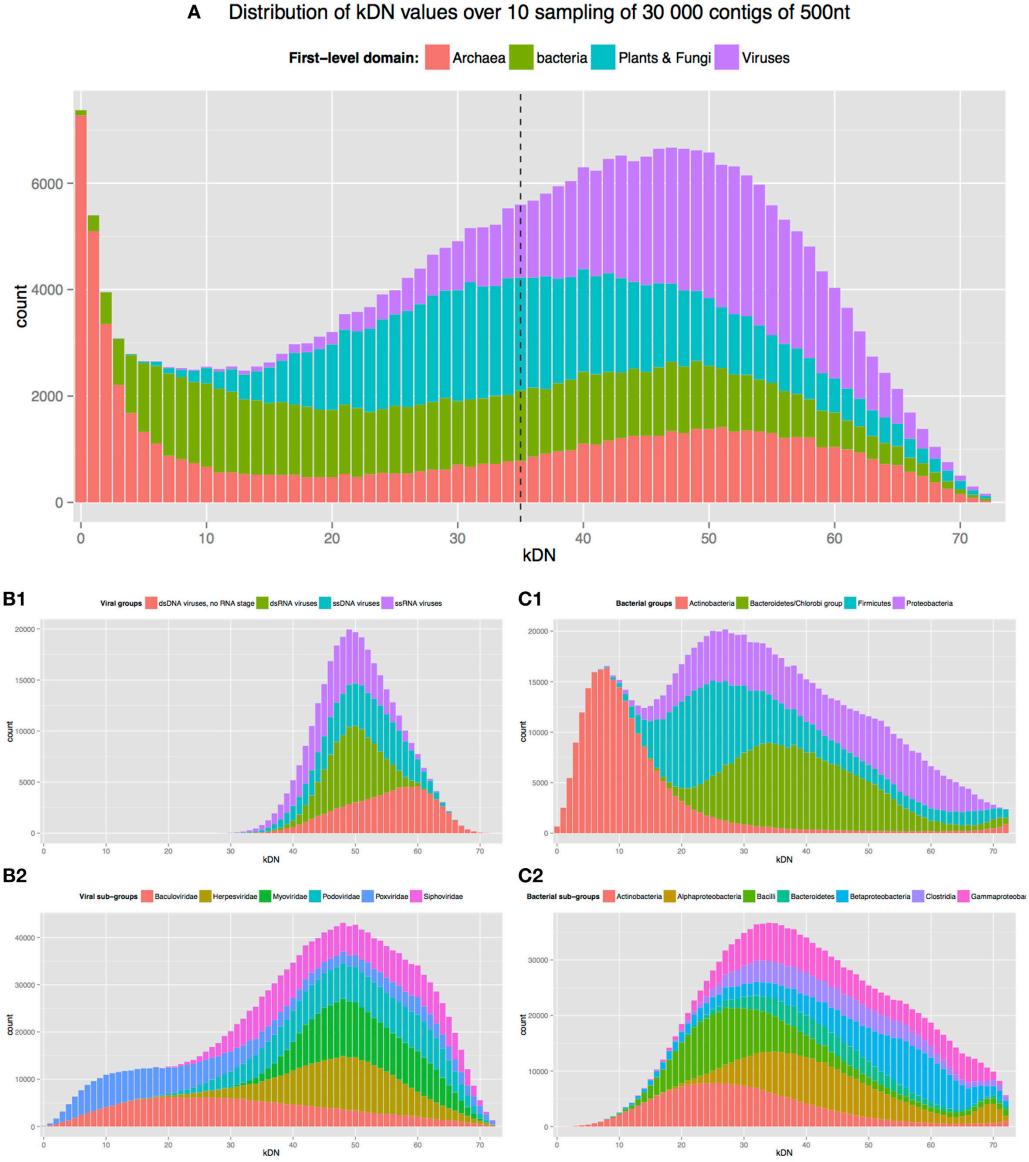
**Distribution of kDN by classes for each of three classification tasks. (A)** Corresponds to Task 3—assignment of 500 nt contigs to first-level domains; **(B1,B2)** to Task 2—assignment of 500 nt viral contigs to a group or to a family, respectively; **(C1,C2)** to Task 1—assignment of 500 nt bacterial contigs to a phylum or to a class, respectively. Each of the 300,000 randomly selected contigs sampled from different first-level domains were represented as vectors of 3-mer frequencies. Histograms indicate how many contigs (y-axis) per class (colors) have a certain number of neighbors (x-axis) not sharing their own class label, within the closest 73 neighbors. Neighbors are determined w.r.t. euclidean distance in the space of 3-mer frequencies (cf. Section Why is the First-level Assignment Problem Hard? of main text). For example, there are more than 6000 different archaeal contigs (red bar) not having a single non-archeal contigs in their closest 73 neighbors (red bar corresponding to 0 kDN). The dashed line represents the boundary between contigs easy to classify correctly with a majority vote (to the left of the line) and hard to classify (to the right). Only the top 4 most abundant classes are shown for **(B1,C1)**; and 6 for **(B2,C2)**.

We summarize in Figure 1 the distribution of kDN by class for each of three tasks. The upper panel shows that for the first-level classification task, archaeal and bacterial contigs can be easily assigned to their respective domain, and that this classification is hard for eukaryotic contigs and even harder for viral ones. When the classification task is restricted to bacteria only (panels C1 and C2), fine-grained classification is not hard at both phylum and class levels. For viruses (panels B1 and B2), fine-grained classification to groups (ssDNA, dsRNA etc.) is hard, while assigning a viral sequence to a family level is easier, though less easy than for bacteria. Using a permutation scheme, we established that the observed kDN value is significantly different from the null kDN values for all but the virus fine-grained classification to groups (data not shown).

Consistent with previous work (Mende et al., 2012; Teeling and Glockner, 2012), we have verified that for contigs shorter than 500 nt, distributions are shifted to the right—which corresponds to a harder classification problem (data not shown); conversely, for contigs longer than 500 nt, distributions are shifted to the left, corresponding to an easier classification problem (see Supplementary Figure 1).

**Figure 2:**
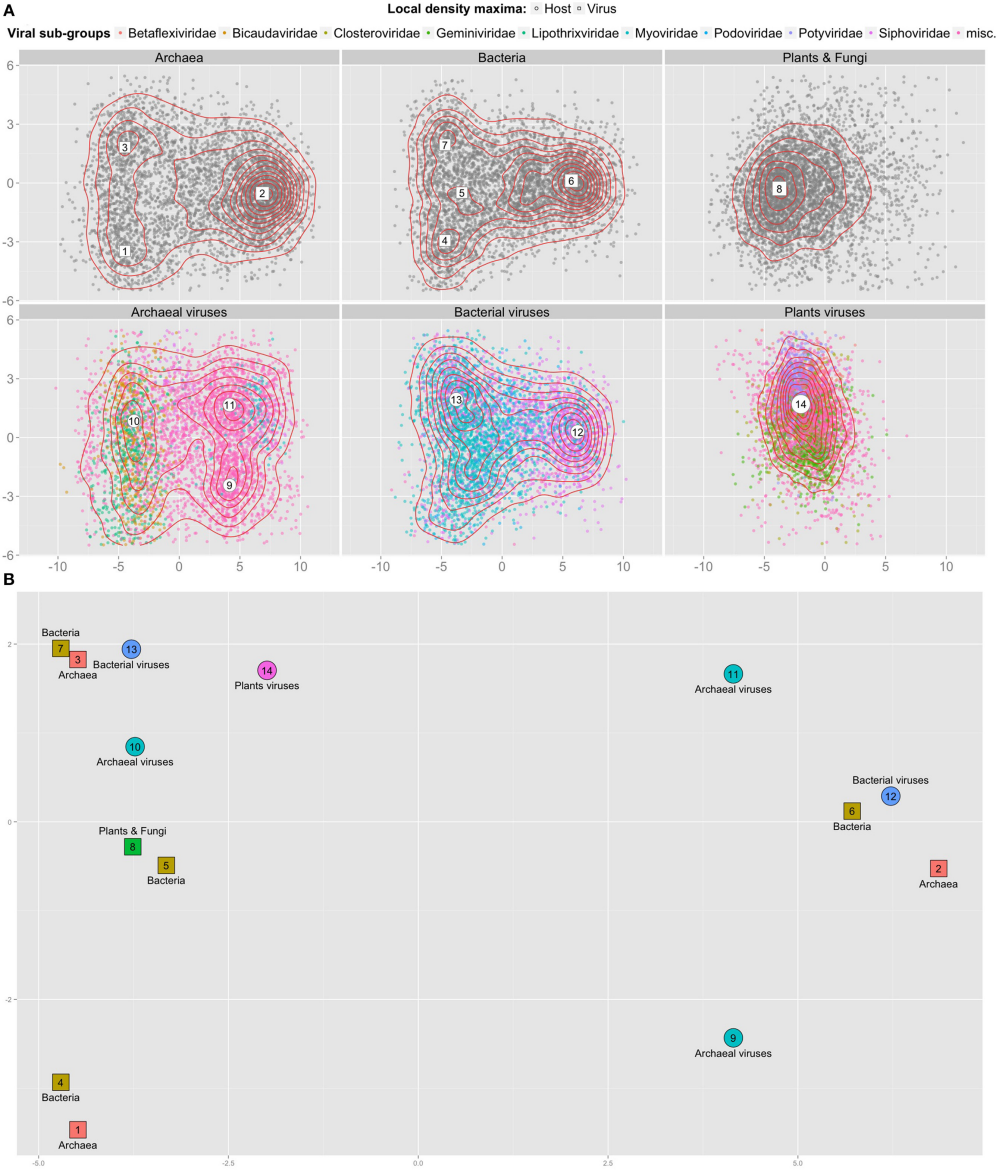
**2D projection of 3-mer frequencies for cellular and viral contigs. (A)** Top two dimensions from the PCA reduction of 28,134 contigs (points) of average length 500 nt represented as frequency vectors of 3-mers; sampled equally from genomes originating from 3 top levels cellular domains (top row) and from 3 viral types known to infect them (bottom row). Dimension 1 (x-axis) accounts for 30% of the variance, dimension 2 (y-axis) for 8% of the variance. For each sub-panel, 2 d kernel density estimation is represented using red contour lines and local density maxima are numbered within large white shapes. **(B)** Close up of **(A)** with all local density maxima. The principal components were computed once for the whole set of contigs of all genomes. Position, coordinates and axes from all sub-panels are comparable.

It has been previously observed that viral 3-mer signatures are close to that of their hosts (Pride et al., 2006). However, evidence contradicting this observation has also been proposed, for example for large viruses (Mraìzek and Karlin, 2007) and for viruses of monocots and dicots (Adams and Antoniw, 2004). We investigated whether the classification difficulty could be explained by overlapping *k*-mer distributions between different types of hosts and viruses that infect them. To this end we sampled 4689 contigs from each first level cellular groups (archaeal, bacterial, plant and fungal genomes); and of viruses known to infect them. Using principal component analysis (PCA), we projected the 3-mers frequencies vectors of these contigs on 2 dimensions. Figure 2 shows that viral and cellular contigs are spread uniformly in these 2 dimensions, with the exception of plants viruses that are more compact. Using local density analysis, we observed that contigs of bacterial viruses indeed are close to their hosts (points 12 and 6, 13, and 7), but that they are also as close to archaeal contigs (points 13 and 3). On the other hand, archaeal viruses are not close to their hosts; while plant viruses are closer to bacteria and archaea than to their hosts.

## Discussion

Distinguishing viral and cellular sequences in non-targeted environmental studies is a yet unresolved classification problem, especially for unknown viral species. We have shown that the reason why this problem has been eluding a satisfactory solution lies in its intrinsic computational difficulty. The reason for this difficulty lies in the fact that viral sequences *k*-mer distributions overlap with cellular one's almost indiscriminately. This is to be contrasted with the relative ease of the corresponding classification task for archaea and bacteria that certainly underlies the success of bacterial taxonomic assignment studies. The difficulty for viral sequence classification will be alleviated as the public sequence databases become further populated with acquired viral data but this will not provide a sufficient solution to the problem of novel species discovery.

We strongly believe that appropriate choice of computational methodology and further research efforts in this direction are key for the advancement of this field. In the current state of knowledge, we recommend adopting the strategy of contig-level assembly of reads combined with k-mer frequency-based analysis for the identification of viral sequences in metagenomic samples. As for the development of new methods, the promising avenue for the discovery of novel viral sequences seems to be the relaxation of the stringency of long *k*-mer indexing.

### Conflict of interest statement

The authors declare that the research was conducted in the absence of any commercial or financial relationships that could be construed as a potential conflict of interest.
