# Supplementary material for: Finding and identifying the viral needle in the metagenomic haystack: trends and challenges
Source: Front Microbiol. 2015 Jan 7;5:739. doi: 10.3389/fmicb.2014.00739 (PMC4285800; doi:10.3389/fmicb.2014.00739)
Supplement: Supplementary file 1 [file Image1.PDF]

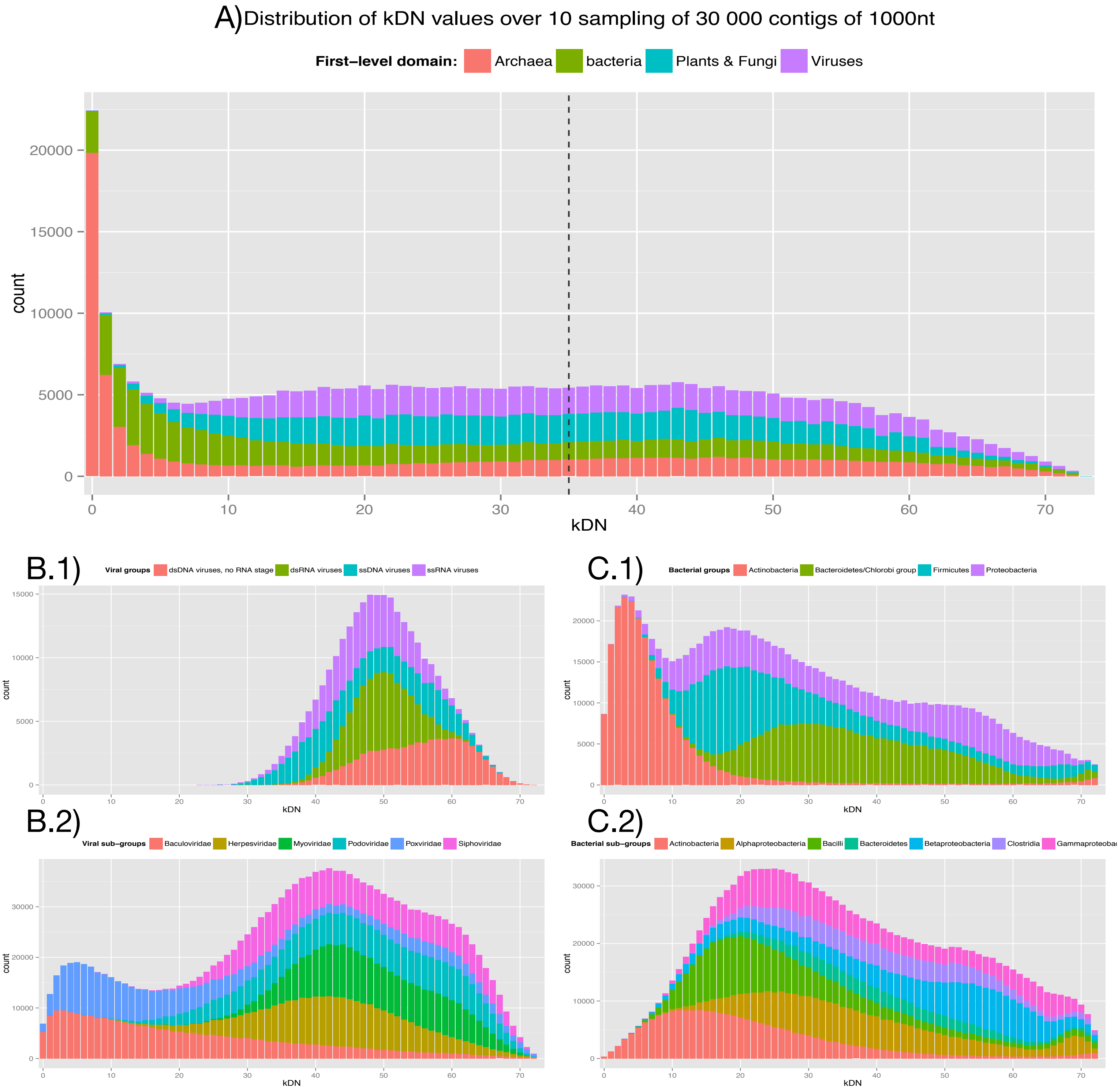

Figure S1 Distribution of kDN by classes for each of three classification tasks for longer contigs (1000nt). Legend and interpretation are identical to Fig. 1 of main text. Panel (A) corresponds to Task 3 - assignment of 1000nt contigs to first-level domains; panels (B1) and (B2) to Task 2 - assignment of 1000nt viral contigs to a group or to a family, respectively; panels (C1) and (C2) to Task 1 - assignment of 1000nt bacterial contigs to a phylum or to a class, respectively. Each of the 300 000 randomly selected contigs sampled from different first-level domains were represented as vectors of 3-mer frequencies. Histograms indicate how many contigs (y-axis) per class (colors) have a certain number of neighbors (x-axis) not sharing their own class label, within the closest 73 neighbors. Neighbors are determined w.r.t. euclidean distance in the space of 3-mer frequencies (cf Sec. 4 of main text). For example, there are more than 20 000 different archaeal contigs (red bar) not having a single non-archeal contigs in their closest 73 neighbors (red bar corresponding to 0 kDN). The dashed line represents the boundary between contigs easy to classify correctly with a majority vote (to the left of the line) and hard to classify (to the right). Only the top 4 most abundant classes are shown for panels (B1) and (C1); and 6 for panels (B2) and (C2).
